# Microneedles: Selected Applications for Pediatrics

**DOI:** 10.3390/children13030360

**Published:** 2026-03-02

**Authors:** Alexis A. Ayala, Hassan Almoazen

**Affiliations:** Department of Pharmaceutical Sciences, University of Tennessee Health Science Center, 881 Madison Ave, Memphis, TN 38103, USA

**Keywords:** pediatrics, microneedles, vaccine, delivery, diagnostic

## Abstract

Microneedles are a pediatric-friendly drug delivery system that has attracted many novel research endeavors. The fact that it creates a tiny cavity in the epidermis, which can allow peptides, proteins, nucleotides, and other large-sized molecules to penetrate the skin barrier, is very advantageous for transdermal delivery. In this review article, we shed light on several pediatric applications of microneedles, such as the delivery of proteins, vaccines, and diagnostics. We believe the advantages of microneedles will continue to expand and be applied to other sites in the body.

## 1. Introduction

Administering medications and vaccines safely, effectively, and painlessly has long been a challenge in pediatric healthcare. Conventional drug delivery methods, such as oral and injectables, are often associated with problems like limited absorption, poor adherence because of poor taste or needle phobia, as well as the need for administration by qualified medical personnel. Microneedle technology has become a game-changer in recent years, providing a painless and effective alternative for pediatric therapy and diagnostics. They do not encounter pain receptors or cause bleeding. These arrays of tiny needles, which are available in solid, hollow, dissolvable, and hydrogel-forming varieties, only penetrate the outermost layers of skin to provide access to dermal microcirculation. Because they have enormous potential as a new frontier in pediatric medicine and the delivery of novel biotherapeutics, they can influence healthcare globally, and this innovation has gained significant attention from both researchers and clinicians. In a variety of pediatric applications, such as vaccination delivery, glucose and biomarker monitoring, local anesthesia, and the management of growth hormone deficiency and infantile hemangiomas, microneedle systems have shown great promise. Microneedles could be regarded as essential in modern therapeutics due to their capacity to deliver drugs with comfort and accuracy. Treatment, diagnostics, and monitoring are all included in the integration of microneedle technology, which has the potential to completely transform pediatric care. Research on microneedle-based biosensors has shown promise for low-invasive, real-time monitoring of children’s glucose and therapeutic medication levels, replacing invasive, painful venipuncture procedures. According to clinical research, children find these technologies less frightening, and parents, along with medical professionals, all have a positive opinion of them. Considering all of these features, children are better at sticking to a regimen. Despite the encouraging results, there are still issues. Regulatory approval, cost-effectiveness, and manufacturing scalability remain obstacles in ongoing research, as does the requirement for age-specific device calibration. However, microneedle-based systems are being investigated as a viable tool for vaccine delivery without cold-chain dependence or resource-intensive personnel requirements, allowing for expanded care to pediatric populations. This is in line with emerging global health initiatives that focus on accessibility and equity, particularly in low-resource settings. This review highlights the increasingly important role of microneedles in pediatric medicine, placing the focus on their therapeutic, diagnostic, and preventive usages. The safety and acceptability of this technology are discussed, covering its economic implications for the healthcare industry. Therefore, this study postulates that microneedle technology is no less than a prototype shift toward more humane, accessible, and sustainable pediatric care.

## 2. Pediatric-Friendly Transdermal Drug Delivery via Microneedles

Sanjukta Duarah et al. performed an exhaustive review on recent developments in microneedles with emphasis on pediatric drug delivery. Their research sought to evaluate the potential of microneedles in overcoming the limitations of conventional transdermal delivery for children, who often experience taste problems with oral drugs, have difficulty swallowing, and are afraid of needles. The review discusses different types of microneedles: solid, hollow, in-coated, dissolving, and hydrogel-forming, with emphasis on their minimally invasive nature and suitability for application in children. One such emphasis in the review is the uniqueness of pediatric skin as a thinner, more permeable version of adult skin. Because of this uniqueness, the careful tuning of the MNS design and the drug dosages and excipient safety are required. The authors acknowledge that despite the potential, there are major challenges; there have not been clinical trials in children, and there is a need for a standard applicator device to ensure safe and consistent dosing. Second, the study reports positive attitudes and favorable responses about MNS use from pediatricians and patients, respectively. It also describes current clinical efforts, particularly for vaccines, such as those against influenza. It concludes with a plea for continued innovation and regulatory support to realize the potential of MNS-based pediatric drug delivery [1].

Delgado-Charro et al. studied the applications of transdermal drug delivery systems in pediatric patients through their review that focused on microneedle technology as an innovative delivery method. The research evaluated TDD safety and effectiveness and feasibility for pediatric use, including preterm infants and neonates, because their skin presents unique challenges for drug absorption. The advanced delivery techniques included microneedles as a promising minimally invasive method to deliver pediatric drug doses across the skin barrier. The safety of microneedles for preterm newborns remains uncertain because their immature skin requires special protection against toxic substances that could penetrate through unregulated absorption. The authors note that most TDD systems were developed using adult skin models, which do not accurately represent pediatric conditions. Research must prioritize the study of infant skin physiology changes and the development of transdermal systems that match their developmental stage. The review demonstrates that there are no approved TDD formulations available for the most vulnerable pediatric subgroups while stressing the need to develop specialized patches and microneedle systems for their unique requirements [2].

The effectiveness of dissolving and hydrogel-forming microneedles in delivering medications through children’s skin was investigated by Caffarel-Salvador et al. The primary goal was to determine whether these microneedles could efficiently administer lidocaine hydrochloride and caffeine, two highly popular pediatric medicines. Their second goal was to find out how the general public, including kids, parents, and medical professionals, felt about this new technology. Developing microneedles that would address the issues of pediatric drug delivery, such as oral drugs in children and injection anxiety, was the primary obstacle the study overcame. Microneedles painlessly pass through the stratum corneum, the outermost layer of skin, without producing bleeding, which is desired by pediatric patients. These microneedles were special because of the way they were made. To increase performance, laser technology was used to create these needles. Through in vitro and in vivo research, the potential effectiveness of these MNS for pediatric dosage was examined. This study demonstrated that polymeric MNS significantly increased the model therapeutic compounds’ skin permeability both in vitro and in vivo. Specifically, hydrogel-forming MNS boosted caffeine delivery by 6.1 times, while dissolving MNS in vitro increased lidocaine HCl distribution by 3.3 times. Rats treated with caffeine-loaded MNS had a 24 h plasma concentration of 23.87 μg/mL. Regarding the possible use of MNS technology in the pediatric population, this study revealed a broad consensus among schools, pediatricians, and the public. Overall, 85.9% of pediatricians and 93.6% of respondents from the public thought that using MNS was a good idea [3].

Alkilani et al. produced a comprehensive review titled “Beneath the Skin: A Critical Review of Current Trends and Future Prospects in Transdermal Drug Delivery Systems.” In this work, they examined potential TDD technologies and assessed their effectiveness as non-invasive methods for drug delivery that are acceptable for patients. The study concentrated on evaluating recent chemical, physical, and hybrid dual-enhancing strategies, particularly delivery systems utilizing microneedles, which are commonly employed to enhance the penetrability of drugs through the skin barrier. Microneedles (MNs), which are considered one of the promising physical enhancement methods for TDD, have been mainly discussed in the review. Although there are different types of microneedles being developed, an interesting exposure from this review is the potential application of sustained-release microneedles with methotrexate nanocrystals. These hybrid microneedles combine the advantages of the particle size reduction in the depth of their skin-penetration capacity with controlled drug release, so that they penetrate the skin deeper and have a longer treatment effect. This makes them highly attractive as potential carriers of drugs that cannot be taken by mouth. However, Alkilani et al. also present various obstacles to microneedle technology. These obstacles could be processing complexity, regulatory issues, and the challenge of large-scale production under GMP. Moreover, designing microneedles using drug-loaded nanocrystals while preserving their structural integrity and therapeutic efficiency is still a formidable challenge [4].

The goal of Sheniqua Brown’s study was to improve glucose tracking in critically ill pediatric patients by developing a method for monitoring transdermal glucose using microneedles. In comparison to the existing glucose monitoring methods, it was hoped that the new approach would be less invasive and less painful for patients. To enhance permeability and enable more passive diffusion of interstitial fluid glucose through the skin, the study employed solid microneedles to attempt to make tiny openings in the skin. These microneedles were special since they just needed to be applied to the skin for around 30 s to start working. In addition to being far quicker than any existing technique, this method does not require hydrogel swelling or vacuum suction for sample collection because it uses a non-extractive PBS-based collection protocol. A very sensitive fluorescence biosensor was utilized to measure glucose. For glucose, this sensor demonstrated micromolar sensitivity and good selectivity, enabling the detection of traces in transdermal samples. Pig skin, which is quite comparable to human skin, was used in multiple lab trials by researchers to test this approach. The amount of glucose that could be collected via the skin was discovered to be enhanced using microneedles. Using vibrating microneedle pens, which occasionally resulted in ineffective skin openings, was the team’s largest problem. To alleviate this variability, a one-press application mechanism may be used in future development. Furthermore, research on adult participants investigating different locations for MN administration could aid in refining this minimally invasive method for use with children [5].

A study was carried out by Xiaokun Lin et al. to create a microneedle for the transdermal administration of timolol maleate to treat infantile hemorrhages. Infantile hemangiomas are common non-cancerous tumors made of blood vessels that appear in infants. The main focus of the study was to design a hydrogel microneedle system made from a protein called BSAMA in order to overcome the limitation of poor drug penetration for current topical treatments. UV light and vacuum technologies were used in the creation of these microneedles to guarantee their correct shape and sharpness. Because BSAMA is a protein that is both safe for the body and powerful enough to pass through the skin, its application is distinct. The medicine can be released gradually thanks to this design. About 69% of the medication is released over the course of 72 h. The ability to remain stable at various storage temperatures is another benefit of these microneedle technologies. The research team found encouraging findings in the mice’s tests, demonstrating that the microneedle design effectively penetrated the mice’s skin. In vivo tests on mice with tumors resembling hemangiomas revealed that the microneedles dramatically suppressed the growth of the tumors and decreased the expression of GLUT-1, a hallmark of hemangioma proliferation. Compared to conventional topical administration, it also reduced the requirement for frequent dosing. One excellent discovery was that these microneedles had no adverse effects or skin damage, demonstrating how safe and efficient this may be for use in children [6].

Li Yang et al. conducted a study developing a microneedle patch designed for the sustained delivery of recombinant human growth hormone to treat growth hormone deficiency in pediatric patients. Recombinant human growth hormone, which shares the same structure and pharmacological properties as endogenous growth hormone, is currently administered as a daily subcutaneous injection as a therapy for growth hormone deficiency. Growth hormone deficiency therapy typically necessitates years of ongoing care, resulting in thousands of subcutaneous injections for patients. Frequent dosage puts a significant financial and psychological strain on patients and results in noncompliance, which produces imperfect results. The main objective was to get beyond the drawbacks of regular subcutaneous injections. The microneedles were developed into a microneedle patch (PAA/NaHCO3-Silk MN) based on silk protein for sustained release of recombinant human growth hormone. In rats, the MN patch could maintain the sustained release of rhGH for more than 7 days and produce similar effects as daily subcutaneous injections. The PAA/NaHCO3-Silk microneedle patch has several advantages, including the potential for painless self-administration and the elimination of the requirement for cold-chain storage and transportation, both of which have significant financial advantages. It was also safe, well-tolerated, and biodegradable under the skin, which made it perfect for kids. The study concluded that actively separated microneedle patches could improve the treatment effect of GHD and provide a new self-management method for GHD patients [7].

Cormier et al. did a study using a coated microneedle array patch system to deliver desmopressin via transdermal. Their aim was to address the limitations of existing desmopressin delivery methods in pediatric use. Desmopressin is a strong synthetic peptide hormone that is primarily used to treat hemophilia A, diabetes insipidus, and enuresis in children. Injectable formulations demonstrate better bioavailability but are poorly suited for routine use in young children. The study used Macroflux technology, and researchers wanted to see the feasibility, efficiency, and tolerability of the system. The technology used titanium microneedles. This made it possible for medications and immunizations to get past the skin barrier. For quick bolus administration, the needles can be coated with medication; alternatively, they can be used in conjunction with a drug reservoir for ongoing passive or iontophoresis treatments. The hairless guinea pig was used to test the technology’s potential effectiveness in administering desmopressin through the skin since its skin anatomy is more like that of humans than rodents. The Macroflux technology had good bioavailability in the guinea pigs, with bioavailability as high as 85%, and showed acceptable variability. These results suggest that transdermal delivery of desmopressin by Macroflux is a safe and efficient alternative to currently available routes of administration [8].

## 3. Microneedles for Non-Invasive Diagnostic Monitoring in Children

Kiang et al. reviewed the literature regarding microneedle-integrated biosensors for therapeutic drug monitoring via interstitial fluid as a viable alternative for conventional TDM via blood draws, particularly in sensitive populations, such as pediatrics, geriatrics, and anemics. The review emphasized the viability of microneedles for low-invasive, painless access to ISF located within the dermis for drug monitoring purposes. The microneedles can conduct drug analysis on the microneedle apparatus itself, decreasing the need to transfer interstitial fluid into external laboratory apparatuses. Two types of devices were reviewed: hollow silicone microneedle sensors and silicon dioxide microneedle sensors embedded with enzymatic or optofluidic biosensors. For two of the TDM devices, successful retrieval and detection of glucose and vancomycin occurred with quantities as little as one microliter or sub-nanoliter ISF. However, the authors contend that many issues remain. Issues include limited ISF retrieval volumes, long durations for ISF retrieval, and difficulties in transporting ISF from one area of the microneedle device to its sensing components. Furthermore, although preliminary findings were promising, neither device has yet undergone a human trial or FDA approval. Ultimately, the article concludes that ISF-based TDM via microneedles has therapeutic potential for real-time, bedside drug-level monitoring in patients requiring antimicrobials or anticonvulsants, but further research in humans is warranted, with additional pharmacokinetic modeling for therapeutic effectiveness [9].

Pires et al. reviewed the possible use of microneedles to treat and possibly track health issues in children. With painless and noninvasive medication administration and biomarker monitoring, the primary objective was to determine how microneedles, namely polymeric, coated, hollow, hydrogel-forming, and dissolvable microneedles, could impact and advance pediatric healthcare. The ability of microneedles to pierce the skin’s outer layer without bleeding or causing significant discomfort is what sets them apart. This trait is especially important in kids who frequently complain and have needle phobias. These microneedles may one day be used to deliver medication and serve as a biomarker detector as technology develops. The authors discuss the fascinating new idea of coupling microneedles with micropumps and biosensors. This technology would eliminate the need for a routine injection by automatically administering the appropriate medication and checking things like blood sugar. Children with long-term conditions like diabetes, asthma, or growth hormone issues may find this to be quite beneficial. The article discusses a number of important issues, including the necessity to customize microneedles to meet pediatric needs because children’s skin is softer and thinner than that of adults. Furthermore, pediatric skin varies by age group and body part, so much customization is still required before this technology is widely used and before it can be considered safe and effective [10].

O’Sullivan et al. conducted a study to evaluate a portable EEG acquisition system for use in neonatal intensive care units, mainly to see the efficiency of dry microneedle electrodes as an alternative to traditional wet electrodes. The focus was to see the feasibility of dry electrodes, which do not require sticky gels or skin scraping, which could be beneficial and safer in a clinical neonatal setting. Two varieties of dry electrodes were put to the test. The first was MicroTips, also referred to as micro transdermal interface platforms, which are ideal for bare skin areas like newborns’ foreheads because they use small needle tips to puncture the top layer of the skin, lowering impedance and enabling signal acquisition without the need for gels. The g.tec electrode, on the other hand, which has bigger pins, worked better on areas with hair, such as the occipital region. Neonatal testing presented ethical and safety issues for the project, which were resolved by using a simulation framework. The researchers employed actual EEG recordings from infants and passed them through a system that replicated the behavior of various electrodes on the skin. This framework allowed for controlled testing of various electrodes without involving human neonates. Additionally, the study compared dry electrodes to conventional wet electrodes through in vivo testing on adult participants. According to the results, dry electrodes were more sensitive to noise and had weaker signals than wet electrodes, but they were still very effective when applied to the forehead with MicroTips and the back of the head with g.tec. All things considered, neonatal care and outcomes will be improved if medical personnel have access to rapidly applied EEG recording equipment that allows them to evaluate the brain both shortly after birth and during suspected abnormal neurological activity. The development and testing of portable, easily navigable EEG technology for the neonatal population are supported by this article [11].

In order to determine how well a novel technique known as “Star Particles” can improve the administration and onset of topical anesthetics in pediatric patients, Tadros and his colleagues conducted the first randomized clinical trial in humans. The goal of the study was to address the drawbacks of topical anesthetics already on the market, which often have a delayed onset because of low skin permeability. Star Particles are a novel type of microneedle composed of titanium dioxide projections shaped like stars. To improve permeability, they were intended to be used in a topical gel. The study examined three common drugs, including lidocaine, epinephrine, and tetracaine gel, and recruited 22 healthy children aged 10 to 15. The children participating in the trial received either ordinary LET gel or LET gel that had been administered following the use of STAR particles. It was noted that the STAR/LET therapy was more effective. It demonstrated increased water loss in the skin and decreased overall pain in children. No adverse effects were noted, and many of the kids said the STAR particle was comfortable and painless. For children undergoing needle-based medical procedures, STAR particles provide a new, easy-to-use, affordable, and well-tolerated way to quickly apply topical anesthetics to the skin for locoregional pain management [12].

## 4. Safety, Acceptance, and Feasibility of Microneedles in Pediatrics

Karen Mooney and her colleagues investigated how kids perceive microneedles as a less unsettling alternative to conventional blood collection in patient monitoring. The purpose of the study was to determine the acceptability of microneedle technology by children between the ages of 10 and 14, particularly for routine monitoring, where discomfort and needle phobia are frequent barriers. Patch-based microneedles, a kind of low-invasion device that can monitor or administer drugs through the skin without drawing blood or causing excruciating pain, were used in the study. Because of their small size, lack of visible needles, and ability to be ornamented with entertaining themes like cartoons, these microneedles were less frightening and more appealing to children. The patch design, which decreased anxiety and procedure awareness, was a significant component of the study. Nevertheless, the study discovered certain difficulties. Youngsters voiced concerns about potential allergic reactions, doubted the technology’s accuracy, and found the word “needle” harmful. Participants emphasized that increasing acceptance requires clear communication and education. They proposed that renaming the technology to omit the word “needle” might alleviate concerns. The potential of microneedle technology for kids is demonstrated by this exploratory study, which also offers helpful insights into how educating and designing with children in mind can promote acceptance. The viewpoints of kids with long-term illnesses who require frequent monitoring warrant further study [13].

Marshall et al. conducted a review of the published literature to evaluate the perception, acceptability, and appropriateness of microneedle technology in immunization and its application in the pediatric age group. However, their study did not include new clinical data; instead, they analyzed clinical results from 12 other studies, out of which eight featured microneedle delivery and four featured technology. The different microneedles considered in the review were solid, coated, hollow, dissolvable, and swellable microneedles. These devices are designed to penetrate only the outer layer of the skin and not to reach the pain receptors or the blood vessels, thus making the procedure painless and not very invasive, a feature particularly appreciated by children, who often develop anxiety from the sight of a needle. The advantage of microneedles, and what makes the technology so interesting, is that it may provide a more simplified and user-friendly delivery system for vaccines, which could reduce the requirement for trained personnel, eliminate hazardous sharps waste, and improve compliance by reducing the need for repeated administrations. Indeed, as Marshall explains, there is a lack of understanding of the potential acceptability of microneedles and of how such methods would be perceived, at least among parents, children, and healthcare workers. However, readers should note that this commentary serves to underscore the enormous potential of microneedles to improve childhood immunization but also emphasizes the need for these delivery systems to be adopted by the public as well as the medical community. The authors recommend a higher level of investigation on provider perception and recommend parent and provider education for incorporation into pediatric care [14].

A qualitative study by Marshall et al. examined the acceptability of dissolvable microneedle-patch vaccinations for pediatric use, with an emphasis on parents’ opinions as important decision-makers about childhood immunization. The study’s primary goal was to find out how parents truly felt about this microneedle technology. The microneedles examined in the study were dissolvable polymer-based patches used to deliver vaccinations painlessly through the skin. One major advantage these patches had over conventional needles was that they eliminated the need for cold storage. They can be stored at room temperature and also eliminate sharp waste, making them easier for parents to use and safer for children. The parents’ uncertainty about this new technology was one of the difficulties associated with the microneedle patches. Their worries included possible allergic responses, uncertainty regarding appropriate distribution, and unfamiliarity with the patches. Some were even concerned that if patches were self-administered without medical supervision, coverage rates might decline. However, the majority of parents concurred that receiving education and instruction from medical professionals would make them feel more at ease when applying the patches, demonstrating the significant influence that clinicians have on vaccine acceptability [15].

Instead of using the usual vaccines currently utilized, Adigweme et al. conducted a study to identify a novel method of administering shots like measles and rubella using dissolvable microneedles. One advantage of the microneedles is that they are so tiny that they do not hurt. In reality, the microneedle patch distributes the immunizations as it melts into the skin. The goal of the study was to determine whether the patch was safer and more convenient than a standard vaccination. An additional objective was to determine whether the patch was effective and whether the body could indeed generate enough antibodies to fend off the illnesses. In the first phase of the investigation, adults were used. The study trial was intended to be an age-de-escalation study, and in order to ensure its safety, it began with healthy adults between the ages of 18 and 40 before advancing to toddlers. The toddlers, who were between 15 and 18 months old, had already had their measles and rubella vaccinations. Infants aged 9 to 10 months who had not yet been exposed to the vaccination made up the final group in the experiment. The fact that the trial was double-blinded and that the study team had to closely monitor side effects due to working with infants and children was among the difficulties encountered. This trial was significant since it was among the first clinical trials to concentrate on pediatric use and employ microneedle technology in children [16].

Research by Karen Mooney et al. evaluated UK pediatricians’ perceptions of the application of microneedle (MN) technology. The primary goal was to determine whether microneedles could be a suitable substitute for conventional blood tests, which children find frightening and uncomfortable. The research team produced polymeric microneedle arrays, which were the microneedles employed in this investigation. In addition to being antimicrobial, these microneedles were designed to be break-resistant. They were also made to be painless and cause less bleeding, which is what most children find frightening about traditional procedures. This study was distinctive since it concentrated on the opinions of healthcare professionals, particularly pediatricians. The high expense and possibility of allergic responses or skin irritations were the main concerns about using microneedles. The training needs for staff, parents, and older children to utilize the technology safely and effectively were the study’s final big worry. Before microneedles are extensively utilized in homes or hospitals, issues like these need to be resolved first [17].

Matthew N. Berger et al. did a systematic review and meta-analysis to evaluate the immunogenicity, safety, and acceptability of microarray patches. This study highlights them as a promising vaccine delivery mechanism that can effectively target the epidermis and upper dermis, which is rich in immune cells. This could potentially increase immune responses. This technology is unique in that it can eliminate sharp waste and allow for self-administration. Just like other microneedles, they do not really need cold storage, which is more cost-effective. The review included 22 studies and showed that microarray patches provided better immunogenicity compared to the traditional methods. In microarray patches, no significant adverse effects were noted. After applying microarray patches, erythema was more common than with needles and syringes, but it was short-lived and well-tolerated. Following microarray patch application, pain scores were typically lower than those for needles and syringes. According to most studies, microarray patches are very user-friendly and well-liked by parents and medical experts. Although more research is required, microarray patches for immunization were found to be safe, well-tolerated, and elicit immunogenicity that was comparable to or better than that of needles and syringes. Because microarray patches cause less pain, vaccine uptake may be increased [18].

Kaaijk et al. conducted a questionnaire study to evaluate Dutch parents’ attitudes toward the amount of vaccine injections per clinic visit and on needle-free vaccine delivery methods for children. For this purpose, the parents’ opinion toward a jet injector, a patch, a microneedle system, and a nasal spray device as methods for vaccine delivery was assessed. After the questionnaire was finished, it was determined that the majority of parents (69% with a score > 4) indicated that three vaccine injections per visit was too much. In the study, parents appeared to prefer to vaccinate their child with a jet injector (M = 6.03, SD = 1.17) or a patch (M = 5.40, SD = 1.69) compared with the conventional syringes. However, nasal sprays and microneedles were not thought to be superior to conventional injections. For older children, parents were more receptive to alternatives, but not for very young ones. According to the current survey, parents are generally supportive of the jet injector and patch as substitutes for traditional syringes in vaccination delivery. This should motivate vaccine manufacturers and developers to work on creating these vaccine delivery systems [19].

Fernando et al. conducted a first-in-human clinical study using the nano patch to deliver an influenza vaccine to overcome limitations of traditional vaccinations, particularly in pediatric populations. The nano patches were manufactured from silicon wafers, dry-etched, and diced to give individual patches of 10 × 10 mm with micro-projection arrays (10,000/cm^2^) of 250 µm in length. They were dry-coated with vaccine and delivered via a spring-powered applicator at high velocity to penetrate the outer layers of the skin. Unlike dissolving microneedles used in previous studies, the nano patch retains structural integrity, enabling a 2 min application without the need for adhesives or extended wear time. One drawback of this microneedle technology was that there were local skin reactions such as erythema and itching, but they were mild or moderate and self-limiting. In this study, the nano patch vaccine delivery method seemed to be safe, well-tolerated by the recipients, and produced comparable immune responses to intramuscular injections. According to the findings, the nano patch may prove to be a successful method of vaccinating against seasonal influenza and other diseases [20].

Guillermet et al. did a study to evaluate the acceptability of a novel vaccine technology called nano patch for pediatric immunization in low-income countries. A nano patch is a solid or dissolvable microarray patch. They are one mm in length, which administers a dry formulation of a vaccine or pharmaceutical. The study gathered its data by collecting opinions from key stakeholders involved in the EPI and vaccine safety and management at both the central and district levels. They also got opinions from caretakers of children aged 9–23 months and community representatives. A total of 314 people participated in the study. The overall acceptance percentage of the child vaccination patch was 92.7%. Overall sentiment was highly favorable, and clinical research showed that microarray patch treatment is safe and beneficial in preventing infectious diseases [21].

## 5. Microneedle Technology for Pediatric Vaccine Delivery, Especially in Low-Resource Settings

V. Kumar and his team conducted a study to see whether microneedle patches may deliver vaccines more safely and effectively in underdeveloped nations with limited access to medical care. The premise is that, in comparison to vaccines, these microneedles would be more effective because they do not require skilled nurses or doctors to administer them, and they cause less pain. The vaccinations were administered through the skin using 3D solid and dissolvable microneedles. The capacity of MNS patches to preserve vaccine stability at higher temperatures is one of its many noteworthy benefits. Vaccines against rubella and measles have been shown to last longer without refrigeration. This feature has the potential to significantly lower the expenses and logistical strain of cold-chain storage, increasing vaccination accessibility in rural locations. These kinds of cutting-edge technologies can aid millions of children in rural regions who lack access to essential vaccinations through microneedle technology [22].

Adigweme et al. conducted a clinical trial evaluating the use of dissolvable microneedle patches for the delivery of the measles and rubella vaccine in pediatric populations. The focus was to look at the safety and immunogenicity of microneedle technology compared to traditional technology. The type of microneedles used in this trial was dissolvable needles embedded with live attenuated MRV. This was designed to be applied painlessly to the skin, where the needles would dissolve and release the vaccine into the dermis. A major innovation of this patch is its thermostability and ease of use, which could enable non-healthcare workers to administer vaccines. This would be optimal in places where there are few resources. One of the main challenges encountered in this trial was skin reaction, specifically indurations and transient pigmentation. These were not serious and went away on their own. In the study, toddlers and infants were randomly given a microneedle or a traditional vaccine. The trials showed that the patch worked just as efficiently as the typical injection. The results showed that 93% developed protection against measles and almost 100% developed protection against rubella. In conclusion, this research presents the first information on the administration of vaccines to infants and toddlers using MNP. The MNP technology was recently listed as the greatest worldwide innovation priority for attaining equity in vaccine coverage in low-income and middle-income nations. The findings made on immunogenicity and safety support the development of this technology [23].

Moon et al. conducted a study using a dissolvable microneedle patch to deliver a combination of inactivated rotavirus vaccine and inactivated poliovirus vaccine through the skin. The aim was for pediatric immunization in poor countries. Their objective was to develop a needleless vaccine delivery method that would be more practical and easier to employ in low-income nations where conventional vaccine delivery is problematic. The team developed two generations of microneedle patches. The first one contained 112 needles made from carboxymethylcellulose, while the second one used methylcellulose. One major concern when combining the two vaccines in one patch is whether they might interfere with each other and weaken the immune response. However, this study showed that both types of microneedles worked efficiently together and had a great immune response in rats. They had no evidence of immunological interference. They also noticed that smaller doses were highly effective, which is ideal to reduce vaccine cost and increase accessibility to more populations. Their results encourage the advancement of this strategy to provide children worldwide with a new combination vaccine that protects against poliovirus and rotavirus [24].

In a study by Moon et al., inactivated rotavirus vaccination was administered through the skin using dissolvable microneedle patches. Microneedles prevent hypodermic needle waste. When compared to IM and SC delivery, microneedles have demonstrated superior immunogenicity and dosage sparing in the administration of several different vaccines. The microneedles were fabricated from stainless steel sheets and coated with IRV using a procedure modified from a published study. One of the main challenges addressed was maintaining the structural integrity and antigenicity of the IRV after coating and drying on the microneedles. They used electron microscopy to evaluate the IRV’s structural integrity. They used rabbit hyperimmune serum to assess the reactivities of TLPs before and after coating by EIA to the human rotavirus strain Wa in order to determine whether eluted IRV retained antigenicity. They found that the absorbance values in both preparations were comparable. Importantly, microneedle coating does not seem to change the structural integrity and antigenic reactivities of the IRV. These findings show that inactivated rotavirus TLPs in coating buffers may withstand drying on microneedles. This study showed, for the first time, that IRV applied topically with a microneedle patch can effectively stimulate a strong immunological response in mice. The creation of an affordable and efficient patch-based IRV may be made possible by these findings [25].

To address ongoing difficulties in measles and rubella vaccination campaigns, Goodson et al. carried out an extensive investigation centered on the use of microneedle-based microarray patches. According to the authors, microneedle-based microarray could play a major role in advancing the Immunization Agenda 2030 goals, such as eliminating measles and rubella, reaching zero-dose children, and expanding vaccination demand, coverage, and equity. They think that these technologies’ lack of thermostability, ability to eliminate sharps waste, ease of administration by community health professionals, and streamlined logistics have made this achievable. In environments with limited resources, this would make them perfect for routine immunizations and large-scale vaccination programs. For example, in the traditional vaccine programs to avoid loss of potency, they must be kept in a continuous cold chain in the dark at 2–8 °C, or −20 °C for long-term storage. If stored in a dark place at 2–8 °C, then the shelf life is 24 months from the date of the last satisfactory potency test. These strict temperature outlines for the current vaccination method are one key factor that prevents it from being more accessible to thousands of people. Much more study needs to be done, including a thorough assessment of the immunogenicity and safety of microneedle technology. Large expenditures and commitments will be required for manufacturing facilities for mass production if it is shown to be safe and effective in order to create a steady and sustainable worldwide supply [26].

Ya-Xiu Feng et al. conducted a comprehensive review on the use of microneedles as a vaccine delivery system to address global challenges of mass vaccination during the COVID-19 pandemic. The main objective of the study was to see how microneedle technology can overcome limitations of traditional vaccination methods, particularly for special populations like children, the elderly, and those in remote areas. The three types of microneedles that were employed were coated, solid, and dissolved. These technologies’ primary benefits are their painless administration and the removal of the need for cold storage, which is often necessary for most vaccinations. Additionally, the ability to self-administer the vaccine would remove the requirement for trained personnel. This would allow for more access and fewer obstacles during hard times, such as a pandemic. However, the administration of the COVID-19 vaccination through microneedles is currently plagued with numerous obstacles. There is significant instability and difficulty in uniformly loading the vaccine during the drying, curing, and loading processes of the microneedles. As a result, the matching antibody titers are unstable. Breakage of the needle tip may provide a safety risk for solid MNS or hollow MNS. Whether in the process of manufacturing or in storage, sterility is difficult to guarantee. Even though there are still issues with technology, manufacturing, and oversight for MNS vaccinations, businesses and researchers are aggressively utilizing emerging technologies to make MNS vaccine injection easier. The world may be better equipped to handle the next pandemic if microneedle vaccine technology is developed [27].

A study by Carlos Wong et al. assessed the cost-effectiveness and possible clinical results of administering the influenza vaccine using microneedle patches to Hong Kong children who have refused intramuscular vaccination. In order to compare the possible outcomes of an IM vaccine program with one that offers microneedle patch vaccines, the researchers developed a decision model. The findings indicate that, in comparison to the standard IM program, the IM/MNP program was more expensive per kid and had lower rates of hospitalization and influenza infection. In conclusion, the WTP threshold, length of sickness in outpatient settings, and MNP vaccination cost all affected the acceptability of the IM/MNP program as the preferred treatment [28].

**Figure 1:**
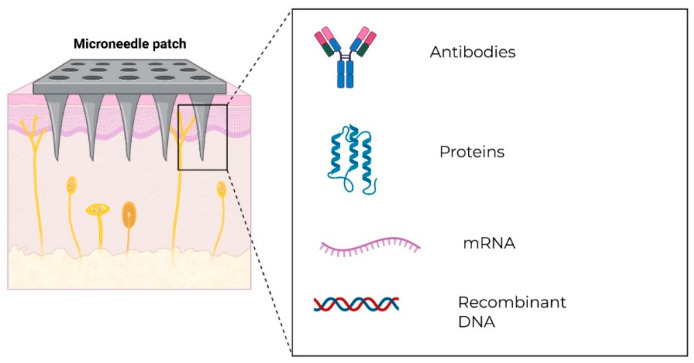
Microneedles can be utilized to deliver different types of novel biological therapeutics (antibodies, proteins, mRNA, and recombinant DNA).

Menon et al. conducted a study using dissolvable microneedles to deliver a nanoparticle-based vaccine targeting the Respiratory Syncytial Virus. The aim was to develop a painless and efficient immunization strategy suitable for pediatric use. To ensure that the needles would dissolve within ten minutes of skin penetration, the team created dissolvable microneedles using hyaluronic acid and trehalose. The RSV fusion protein’s virus-like particles were encapsulated in PLGA nanoparticles using the needles (see Figure 1). Long-term antigen release and enhanced immune cell absorption were made possible by this technique. One of the main challenges was to create a microneedle strong enough to penetrate through the skin and, at the same time, dissolve quickly for efficient antigen delivery. The hyaluronic acid combined with trehalose achieved the best balance of strength and dissolvability. Heightened induction of CD8+ T cells was observed in both the lymph node and spleen cells of the mice vaccinated with the F-VLP NP + MPL NP MN compared to the unvaccinated naïve mice and the mice vaccinated with the F-VLP suspension MN. The induction of CD4+ cells was significantly higher in the lymph node cells of the mice vaccinated with the F-VLP NP + MPL NP MN when compared to the naïve mice and the F-VLP suspension MN group. These results are promising for pediatric vaccination, as the dissolving microneedles provide a minimally invasive and pain-free alternative to traditional injections. The study concluded that PLGA NPs of F-VLP loaded in dissolving microneedles can be a potential vaccine candidate for RSV [29].

Hand, foot, and mouth illness is an acute enterovirus infection that typically affects newborns and young children. It is characterized by fever, mouth ulcers, and vesicles, especially on the palms and soles. Zhuangzhi Zhu et al. did a study using dissolvable microneedles to deliver EV71 virus-like particles as a vaccine for pediatric use for this disease. The majority of vaccinations, including inactivated EV71, are given by injection either intramuscularly or subcutaneously. These methods have several disadvantages, including the need for medical professionals, pain, and cold chains for distribution and storage. The microneedles utilized in this investigation were biocompatible and able to retain their structural integrity in humid environments since they were made with sodium hyaluronate. The main challenge faced in this study was to determine if the microneedles could keep the structural integrity of VLPs. They used TEM and DLS to compare VLPs before and after encapsulation in order to determine if the encapsulated VLPs retained their structural integrity during the drying process. The results showed that encapsulated EV71 VLPs maintained structural integrity during the room-temperature preparation. According to studies on mouse vaccination and virus challenge, microneedle immunization produced high levels of antibody responses that provided complete protection against the deadly EV71 virus challenge. These responses were similar to those obtained from traditional intramuscular injection, but only one-tenth of the antigen was administered. In conclusion, dissolving microneedles might be a viable and successful transcutaneous vaccination method for children’s HFMD prevention [30].

Koutsonanos et al. did a study using microneedle patches to deliver an influenza subunit vaccine. The main objective was to enhance immune protection in pediatric populations. The solid microneedles were composed of vaccination antigen-coated stainless-steel structures. The naturally lower immune response seen in young children because of their immunological immaturity was one of the main issues the study addressed. To see the effectiveness of influenza vaccination in children, they measured the levels of functional antibody titers against the hemagglutinin antigen of the influenza virus induced after the skin delivery with microneedles or intramuscular immunization. No significant responses were measured on day 14. Young mice that received influenza subunit vaccine through the skin using microneedle patches demonstrated significantly higher HAI titers at day 28 post-vaccination when compared to IM-immunized young mice. In addition to providing the opportunity for additional pediatric immunizations, the study team viewed this skin immunization as a highly promising vaccination approach that will enhance influenza-related outcomes across all age groups [31].

Adhikari et al. conducted a study looking at the cost-effectiveness of using microneedle patches for delivering measles vaccines to children. The main aim was to address the logistical and financial challenges associated with traditional subcutaneous injection methods. In order to calculate the vaccination costs for utilizing syringe-and-needle and microneedle patch technologies, they created a basic spreadsheet model. Additionally, they made use of historical data on measles incidence in communities with poor vaccination rates. They calculated that the first dosage of the microneedle vaccination would cost US$0.95, while the first dose administered by subcutaneous immunization would cost US$1.65. In conclusion, using microneedle patches could save expenses; nevertheless, the cost-effectiveness of the patches would rely on how well they work compared to the current conventional vaccine administration system and how well the recipients accept the patches [32].

## 6. Microneedle-Based Topical and Transdermal Drug Delivery for Non-Vaccine Pediatric Treatments

Shah et al. conducted a comprehensive mini-review on the potential of needle-free and microneedle technologies in the delivery of disease-modifying antirheumatic drugs (DMARDs) in infants and children, including those with juvenile idiopathic arthritis (JIA) and other rheumatic diseases. The primary objective was to address the inadequacies associated with conventional parenteral drug administration in children, with the latter method often manifesting in non-compliance, pain, and emotional distress. The microneedle technology described has the unique capability to deliver macromolecules such as fusion proteins and monoclonal antibodies directly into the viable epidermis (see Figure 1). However, the application of microneedles for the delivery of biologics has not been well studied, which becomes a major challenge and research gap. Additionally, the use of these technologies is also limited due to factors such as skin maturity, drug bioavailability, safety issues, and controlled release in young children [33].

James J. Norman and his colleagues conducted a study to see whether hollow microneedles, in particular, could be used to administer insulin intradermally to children with type 1 diabetes. In comparison to standard insulin pump catheters, the primary objective was to determine whether microneedles could lessen discomfort and increase the rate of insulin absorption. In two different approaches, 16 children in the research received rapid-acting lispro insulin. The conventional method was used one day, while the microneedle was used on a different day. In comparison to the conventional method, the microneedle was found to produce less pain and actually had a faster insulin onset of 22 min and a faster offset of 34 min. Children benefit from this since many children with diabetes are afraid of needles and dislike the discomfort that comes with using insulin needles on a daily basis. This kind of technology might make the process quicker and cause less discomfort, which would encourage children to stick with their treatment more [34].

A retrospective cohort study was carried out by Zhou et al. to evaluate the pharmacoeconomics, safety, and effectiveness of three popular treatment approaches for pediatric alopecia areata: oral tofacitinib, microneedling (MN), and traditional therapy. The Severity of Alopecia Tool (SALT) was used to assess treatment outcomes for 24 Chinese pediatric patients with AA subtypes, ages 2 to 14. The unique aspect of the microneedling method utilized in this study was the use of 31G needles (0.35 mm depth) to produce controlled scalp micro-injuries, which improved the transdermal absorption of herbal extracts like Panax ginseng root extract and Lavandula angustifolia oil. This method demonstrates a new, minimally invasive technique meant to stimulate hair growth and modify the local immune response. The MN approach was innovative but had drawbacks, such as being more expensive and time-consuming than other treatments. Additionally, overall efficacy did not outperform traditional treatment, and 42.86% of MN patients were non-responders. However, MN showed promise as a substitute for cases that were severe or refractory. With a 100% SALT100 response rate, the study discovered that traditional topical therapy, which includes minoxidil and mometasone furoate, is the most successful for children who have just received a diagnosis or are not yet receiving treatment. Tofacitinib, on the other hand, may be more appropriate for severe, long-term cases, despite its variable efficacy, though its systemic effects raise safety and monitoring concerns. The lack of a control group, retrospective design, and small sample size are some of the study’s shortcomings that could limit generalizability and introduce bias. To validate these results, larger studies are required [35].

A clinical trial was created by Muhammad Irfan Abdul Jalal and his research team to see if a maltose-based dissolvable microneedle patch could lessen the discomfort that children with thalassemia experience during routine intravenous blood transfusions. This patch was used in conjunction with a numbing cream (EMLA^®^) to test if it would be more effective than the cream alone. Maltose is a sugar that was utilized to make microneedles. This biocompatible and dissolvable sugar promotes medication permeability while reducing invasiveness, such as minor cuts or pain, by forming tiny pores or microspores in the skin. This study was unique since it was among the first to examine the use of dissolvable microneedles to reduce pain during pediatric IV cannulation, particularly in children with thalassemic conditions. It was also special because it was integrated with ways to measure pain, one of which was the subjective VAS score, also known as the visual analog scale, where pain is measured from a scale of 1–10, where one side is no pain, and the other is the worst pain possible. The other was more objective, which used a device called a pain monitor to measure pain by changes in sweat in the skin. The study encountered several difficulties, including differences in pediatric patients’ perceptions and reports of pain and skin alterations brought on by thalassemia that make intravenous procedures more difficult or uncomfortable. Through the use of microneedle technology, the study aimed to fill a significant gap in our understanding of how to treat pediatric pain and may facilitate faster and simpler IV treatments for children [36].

To investigate less unpleasant alternatives to the conventional palatal injection used for dental anesthesia, Babakurd and his team carried out clinical research. The goal was to determine whether EMLA cream applied to microneedle patches would be a superior choice. Children aged 7 to 11 were the subjects of the trial, which sought to determine whether this new delivery method could lessen discomfort during primary tooth extraction. They made patches of dissolvable microneedles and intended to enhance the EMLA cream’s transdermal absorption via the roof of the mouth. This study was groundbreaking since it was among the first to use microneedle technology in a clinical setting to deliver an anesthetic in the mouth cavity. Ninety children participated in this study and were divided into three groups at random. The first group received a routine palatal injection as a control. The second group used EMLA cream, and the last group used EMLA cream in conjunction with microneedle patches. Three distinct stages of pain measurements were done. The first occurred during the anesthetic, followed by the dental tool probing and, lastly, the tooth extractions. The findings showed that, in comparison to the other two groups, children in the third group, which received both EMLA and microneedles, experienced noticeably less pain during the anesthetic phase. Later on, though, the children’s responses to each of the three groups were remarkably identical, and the pain difference was negligible. One major obstacle to the study is that the thick tissue on the roof of the mouth makes it less absorbent, which reduces the effectiveness of the EMLA cream. By creating tiny holes in the tissue, the microneedles increase absorption and boost the effectiveness of the cream. This study demonstrated that dental operations could benefit from the use of microneedle patches [37].

A retrospective study by Kubiak et al. investigated the utility of percutaneous collagen induction therapy as a supplemental treatment for scar development in children following thermal injury. This retrospective analysis was conducted between July 2013 and February 2016 and involved the completion of 99 PCI procedures for the creation of scars after thermal burns in 47 children and adolescents. A medical roller with 2.5 mm needles, known as a dermaroller, was employed. Every operation was performed under general anesthesia. After the procedure, vitamin A and C oil were applied topically. The dermaroller stimulates wound healing and collagen remodeling by inducing regulated micro-injuries in the skin. This novel procedure is special because it does not remove skin; instead, it encourages the skin to repair itself from the inside out, improving the appearance of scars by making them softer and less elevated. The necessity of general anesthesia for children was one of the study’s challenges, but all 47 kids performed well and displayed improvements in their scars. The toolkit for treating scars from thermal burns in kids and teenagers has been expanded with percutaneous collagen induction. It is advised to conduct more prospective research on the best time to provide this therapy and its long-term effects on children [38].

Dreher and his team carried out a lab-based study to evaluate a novel method for augmenting Z-Plasty tendon lengthening as a first step to clinical translation for pediatrics. In pediatric orthopedics, this is a routine operation for ailments like clubfoot and cerebral palsy. A bovine flexor tendon model was used in the ex vivo investigation to examine novel mechanical augmentation techniques following tendon lengthening via Zplasty. They discovered that the suture repair was successfully enhanced by the needle-interlocked scaffold, which demonstrated an ultimate failure force that was more than double that of the controls. According to the study’s findings, using an interpenetrating biomaterial scaffold is a promising new strategy for enhancing the biomechanical characteristics of tendons [39].

Ploin et al. conducted a study using ultrasound echography to measure skin thickness in children to better design and use microneedles for intradermal vaccine delivery in pediatric populations. The group did this because the skin’s dermis and epidermis are now understood to be immune system components and have been suggested as target tissues for vaccine delivery in order to improve immune responses and utilize fewer vaccination doses. One of the key characteristics of this study is the use of non-invasive ultrasound imaging to generate accurate, child-specific data. They found that the supra scapula’s skin was a little thicker than the deltoids. Additionally, they discovered that the skin on the upper back was noticeably thicker than the skin on the lower back. They ultimately concluded that determining skin thickness enhances the effectiveness of intradermal vaccinations. However, these differences were negligible and did not substantially correspond with age, sex, or BMI, indicating that a single microneedle length would work well for most pediatric patients and injection sites [40].

Ryu et al. conducted a study to test a new way to treat warts, which was to use a bleomycin-coated microneedle patch. The main goal was to find a more efficient way to treat warts compared to the traditional cryotherapy, which at times can be painful to people and cause side effects such as blisters and skin color changes. These side effects are more predominant among the pediatric population. The study used solid microneedles made from polylactic acid coated with bleomycin. These microneedles were designed to dissolve the drug into the skin with minimal discomfort, making them suitable for children and individuals unable to tolerate cryotherapy. Two patients who had more than two wart lesions were included in the study. Cryotherapy and micro patches were the two treatment techniques that were applied at random to each patient’s various warts. The Patient’s and Physician’s Global Assessments were used to evaluate the effectiveness of the treatment. According to the data, the mean PGA and PaGA scores did not alter substantially between cryotherapy and bleomycin microneedle patch treatment. The study also demonstrated that the bleomycin microneedle patch was substantially less painful to use than cryotherapy and that its effectiveness was on par with traditional cryotherapy. To sum up, a bleomycin microneedle patch may be a novel, non-invasive, secure, and successful wart treatment option [41].

Hua Jiang et al. conducted a study using a dissolvable microneedle patch to deliver timolol maleate transdermally for the treatment of hemangiomas to provide a more effective alternative for pediatric patients. The low transdermal drug delivery rates of current therapies, particularly timolol maleate in the form of hydrogels and eye drops, result in longer treatment durations. The study developed a soluble microneedle patch using dextran as the primary component to create microcatheters for the long-term administration of timolol maleate in order to overcome this difficulty. Following the biodegradable microneedles’ penetration of the stratum corneum barrier, the needle tips dissolve in the skin to create microcatheters, which allow for the continuous and painless delivery of medications to the dermis. The study prepared dextran microneedles and dextran/oxidized cellulose microneedles from 20 % dextran solution and 10 % oxidized cellulose/20 % dextran solution, respectively, through PDMS molds. At the end of the study, they developed a topical transdermal drug delivery microneedle patch for treating hemangiomas with β-blockers and vascular embolization. Using a microneedle mold, they created pyramid-shaped dextran/C-cellulose microneedles. They observed that dextran/C-cellulose microneedles had a breaking force of 0.27 ± 0.18 N/needle and that drug-loaded microneedles were able to pierce pig skin they utilized as a reference. In a mouse hemangioma model, the study showed that this method not only significantly reduces tumors but also offers a more practical, efficient, and non-invasive therapeutic alternative [42].

Infantile hemangioma is the most common benign vascular tumor that occurs in infants and young children. Geng et al. conducted a study exploring a new strategy for infantile hemangiomas using a dissolvable microneedle system designed to combine photothermal therapy and ferroptosis. Currently, surgery, laser therapy, and medication therapy are the main clinical treatment options for hemangiomas. Even though laser therapy and surgery can produce beneficial therapeutic results, they can be somewhat expensive, and surgery can leave tissue defects or scars behind. Consequently, non-invasive therapy has steadily emerged as the favored therapeutic approach. In this study, they unveiled a novel photothermal patch that used dissolvable microneedles and was intended for infantile hemangioma treatment. The microneedles had a special coating called TA/FE nanocomplex that helped the drug release in the acidic environment of the tumor. It also combined two therapies, which were photothermal therapy and ferroptosis, which is a type of cell death triggered by iron. The design was used to damage the blood vessels that fed the tumor. The study showed through in vivo experiments with mice that microneedles effectively reduced tumor growth by using both therapies. The authors believe that the innovative concept of “ferroptosis-mediated anti-angiogenic therapy” may hold significant potential for clinical applications in hemangioma treatment [43].

Sayami Ito et al. conducted a study to evaluate the safety and efficacy of a hydrophilic gel patch for epi-cutaneous immunotherapy targeting milk allergy, which is very common among the pediatric population. MPC-specific antibodies were evaluated when milk protein concentrate containing HG was applied to skin that either retained its barrier function or developed puncture holes using a microneedle. Patients with severe milk allergies participated in the clinical investigation. No specific immune response was observed, and it was suggested that MPC contained in HG had immunogenicity and could mean the milk protein was not effectively absorbed through normal skin. However, when small puncture holes were made in the skin using microneedles, the body did produce antibodies. This showed that the milk protein in the patch could stimulate the immune system (see Figure 1). There were no systemic side effects observed in the study. The authors concluded that EPIT using HG is a safe method to enable oral administration even in patients with severe milk allergies [44].

Berzosa et al. did a study utilizing dissolvable microneedles to administer a recombinant, non-toxic B subunit of the heat-labile enterotoxin (rLTB) from Enterotoxigenic Escherichia coli, aiming to create a vaccine approach suitable for pediatric use. The research team created dissolvable microneedles from Gantrez AN-119, which is a polymer that has great dissolvability and biocompatibility. Gantrez AN-119 has also been shown to be non-cytotoxic and has immunoadjuvant properties. The needles were designed to dissolve within 15 min after application. One of the biggest issues the research team encountered was that the development of an effective vaccine against ETEC poses a great challenge due to its pronounced genetic and antigenic variability. However, mice showed encouraging results. Both male and female BALB/c mice received a single dosage of 5 μg·rLTB in MNs via the skin of their ears, which resulted in a large amount of anti-LT IgA antibodies in their feces. Additionally, they noticed that the inoculated female mice’s spleen cells produced more IL-17A, suggesting a mucosal non-inflammatory and neutralizing mediated response. By offering a painless and scalable vaccine delivery method, this microneedle technology could overcome clinical barriers in global child immunization efforts against ETEC-related diarrheal disease. Currently, pharmaceutical companies are working actively to develop microneedle products at an industrial scale. These recent developments are showing that the development of microneedle-based vaccine products at an industrial scale is feasible [45].

Pei et al. conducted a case study to explore the effectiveness of microneedling combined with compound betamethasone in treating severe alopecia areata in pediatric patients. In this report, the researchers used a minimally invasive microneedle infiltration device that allows precise delivery of therapeutic agents through microchannels in the skin. This technique enhances drug absorption and stimulates hair follicle regeneration through the Wnt/β-catenin signaling pathway. What made this study very unique is that they had the youngest patient recorded to date using this technology. The patient was 3 years old. After 6 months of treatment, the patient’s condition was significantly improved, and most of the primary hair loss areas had hair regeneration. Some complications with microneedle use were transient erythema and hyperpigmentation after inflammation, but no permanent side effects were noted. Their study’s primary weakness is that it was a case report, and the follow-up period was brief. The effectiveness and safety of microneedling therapy were only observed in a brief period of time in this investigation. This patient will need to be followed up on in the future, and numerous multicenter, double-blind, randomized controlled trials will be needed to prove the long-term safety and effectiveness of microneedle therapy [46].

Shakya et al. did a study using coated microneedles to develop a minimally invasive and painless method for delivering a preventive allergy vaccine for pediatric use. The research introduced a vaccination approach using stainless-steel microneedles coated with ovalbumen. The ovalbumen was used as a model allergen, and CpG oligonucleotide was used as an adjuvant. The uniqueness of this microneedle system is that it can consistently deliver a significant portion of the allergen into the skin, inducing a robust immune response. The response is comparable to traditional subcutaneous immunotherapy. However, the main issue with subcutaneous injections is that it is painful for children. The good thing, though, is that the study demonstrated that the microneedle CIT platform overcame these limitations and prevented allergic sensitization and airway inflammation in a mouse model. This study impacts pediatric allergy prevention by offering a painless method to vaccinate children who are at risk of developing allergic diseases. Although additional research is necessary to address the long-term potency and safety concerns of MNs-CIT, this study establishes the groundwork for future studies on microneedles in the development of allergy vaccines [47].

## 7. Rationale and Perspective on the Future Use of Microneedles in Children and Pediatrics

**Figure 2:**
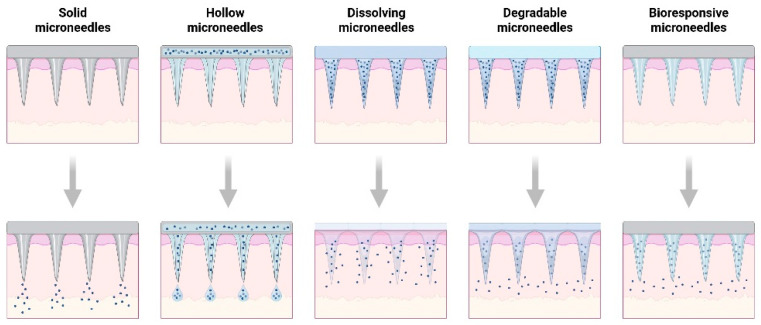
Different types of microneedles are based on drug release mechanisms. The colors indicate the different skin layers: epidermis in pink, dermis in orange, the fat and muscle layers in yellow. The arrows indicate different mechanisms of drug release. The dots indicate the drug release mechanism from the microneedles. The solid microneedles, the drug is coated on the microneedles, the hollow microneedles the drug goes through a channel in the needles, the dissolving microneedles the polymers in the microneedles dissolve and release the drug, the degradable microneedle the polymers in the microneedle degrade and release the drug through pores and the bioresponsive microneedles the drug is released upon trigger due to a binding of an endogenous substance.

There is no doubt that microneedles will continue to identify new and novel applications in children and pediatrics; however, some potential challenges or barriers remain that may influence their use in this patient population. The first barrier is the skin structure itself. The skin structure in children and pediatrics is in the development phase and is less resistant to permeation compared to adult skin. Different skin thickness can affect the microneedle type used based on the microenvironment pH, the drug release profile, and the pharmacokinetics of the drug. The length of the epidermis in children is less than 150 µm and requires thinner microneedles to not disturb the nerve endings. The pH in pediatrics is neutral, which is not very favorable for many salts of basic compounds and can limit solubility or permeability. There are fewer elastin fibers, less fat, and fewer corneocytes and keratinocytes compared to adults, which provides less barrier for drug skin penetration and a softer cushion for microneedles. Children’s and pediatric skin has more moisture than adults, which can dissolve microneedles much faster than in adults and can influence the type of microneedles used. There are several types of microneedles in the literature based on the location of the drug within the needles and their drug release mechanism. There are solid microneedles that can increase the permeability of the drug by creating microincisions or holes inside the skin. A separate patch is attached on top of the incisions to let the drug diffuse through the skin. In coated microneedles, the drug is embedded in the surface coating of the microneedles. The drug undergoes rapid release and dissolution from the coat and diffuses into the skin. There are hollow microneedles that create an incision through the skin and release a drug following active infusion or diffusion of the drug formulation through the needle channel. There are the hydrogel-forming microneedles (dissolving or degradable microneedles) that absorb the interstitial fluids from the skin tissue, which causes swelling of the microneedles and induces diffusion of the drug through the swollen microneedle projections. The dissolvable microneedles lead to a rapid or controlled release of the drug that has been incorporated within the microneedles. The degradable microneedles are another type of gel forming microneedles where the polymer degrades and releases the drug. There is a new type of microneedles that respond to physiological signals called responsive microneedles. One example is glucose-responsive microneedles that release insulin once glucose concentration increases in the dermis See Figure 2 for illustration of different microneedles [1,48,49,50]. Currently, there are no FDA-approved microneedles on the market. Most microneedles are in clinical studies.

## 8. Conclusions

The applications of microneedles in children and pediatrics are constantly growing. In this review, we shared some of the recent deliveries of small molecules and proteins to treat diseases in children and pediatrics. The introduction of responsive microneedles is novel and will attract more research attention in the future. There are still no FDA-approved microneedle patches on the market, but soon this may change.

## Data Availability

No new data were created or analyzed in this study. Data sharing is not applicable to this article.

## Abbreviations

MNS	Microneedles
TDD	Transdermal Drug Delivery
TEM	Transmission Electron Microscope
DLS	Dynamic Light Scattering
MNP	Microneedle Patches
MRV	Microneedles with Attenuated Rubella Virus
IM	Intramuscular
SC	Subcutaneous
IRV	Inactivated Rotavirus Vaccine
TLP	Triple-Layered Particles
EIA	Elisa Testing Reader System
WTP	Willingness to Pay
PLGA	Poly Lactic Glycolic Acid
F-VLP	Virus-Like Particles of the RSV Fusion Protein
MPL	Mono phosphoryl lipid
EV71	Enterovirus 71
HFMD	Hand, Foot, and Mouth Disease
DMARDs	Disease Modifying Antirheumatic Drugs
JIA	Juvenile Idiopathic Arthritis
SALT	Severity of Alopecia Tool
PGA	Physicians Global Assessment
PaGA	Patient Global Assessment
